# Non-Apoptotic Programmed Cell Death in Thyroid Diseases

**DOI:** 10.3390/ph15121565

**Published:** 2022-12-15

**Authors:** Feihong Ji, Xinguang Qiu

**Affiliations:** Thyroid Surgery, The First Affiliated Hospital of Zhengzhou University, Zhengzhou 450052, China

**Keywords:** non-apoptotic programmed cell death, thyroid disease, apoptosis, ferroptosis, pyroptosis, autophagic cell death

## Abstract

Thyroid disorders are among the most common endocrinological conditions. As the prevalence of thyroid diseases increases annually, the exploration of thyroid disease mechanisms and the development of treatments are also gradually improving. With the gradual advancement of therapies, non-apoptotic programmed cell death (NAPCD) has immense potential in inflammatory and neoplastic diseases. Autophagy, pyroptosis, ferroptosis, and immunogenic cell death are all classical NAPCD. In this paper, we have compiled the recent mechanistic investigations of thyroid diseases and established the considerable progress by NAPCD in thyroid diseases. Furthermore, we have elucidated the role of various types of NAPCD in different thyroid disorders. This will help us to better understand the pathophysiology of thyroid-related disorders and identify new targets and mechanisms of drug resistance, which may facilitate the development of novel diagnostic and therapeutic strategies for patients with thyroid diseases. Here, we have reviewed the advances in the role of NAPCD in the occurrence, progression, and prognosis of thyroid diseases, and highlighted future research prospects in this area.

## 1. Introduction

### 1.1. Thyroid Disease

The thyroid gland is an endocrine organ, similar in shape to a butterfly, that secretes thyroid hormones [1]. Thyroid disorders are a general term for disorders in which the function, size, and structure of the thyroid gland are altered. Thyroid disorders are common in endocrinology and include goiter, thyroiditis, thyroid nodules, thyroid cancer (TC), hypothyroidism, and hyperthyroidism.

As the most common endocrine malignancy [2], TC incidence has increased rapidly in the last few decades [3,4]. TC can be classified as papillary thyroid cancer (PTC), follicular thyroid cancer (FTC), medullary thyroid cancer (MTC), and anaplastic thyroid cancer (ATC). TC develops from two cell types: PTC, FTC and ATC originate from follicular cells that line the colloidal follicles and are responsible for thyroid hormone biosynthesis and iodine uptake [5,6]. Parafollicular cells (also called C cells), the origin of MTC, are another cell type of the thyroid gland responsible for synthesizing and secreting calcitonin hormones [7,8,9]. Among these, PTC and FTC, which account for 90% of all cases, have a survival rate of 90% and an overall good prognosis [9]. However, a small proportion of patients, such as those with invasive, or metastatic types of cancer, have a poor prognosis [10,11]. In addition, as one of the most aggressive human malignancies, the post-diagnosis median survival of patients with ATC is approximately 3–5 months [12]. In addition to conventional surgery, several therapies exist, such as radioactive iodine (RAI, ^131^I) [13] and tyrosine kinase inhibitors (TKIs) [14]. However, these treatments have limitations, including drug and radioactivity resistance and adverse side effects [10]. Therefore, exploring new treatments for TC remains a current research priority in the discipline.

As a typical organ-specific autoimmune disease, autoimmune thyroiditis (AIT) is the leading cause of hypothyroidism, with a population prevalence of approximately 1–5% [15,16]. Graves’ disease (GD) and Hashimoto’s thyroiditis (HT) are the two most common autoimmune diseases [15,17]. AIT pathogenesis involves complex interactions between genes and the environment. Although HT and GD have opposite clinical manifestations, they share a common etiology of a decreased tolerance to thyroid autoantigens [18]. As a non-neoplastic disease of the thyroid, AIT has also been the focus of academic research. The mechanistic exploration of AIT has led to a greater understanding of thyroid autoimmunity, which in turn has allowed us to identify new therapeutic targets and facilitate the exploration of new treatment options [19,20].

However, the factors affecting thyroid disease remain unclear. Research on the factors influencing its occurrence is of great significance for preventing and controlling thyroid disease.

### 1.2. Non-Apoptotic Programmed Cell Death (NAPCD)

The Nomenclature Committee on Cell Death (NCCD) classifies cell death into programmed cell death (PCD) and accidental cell death (ACD), defining and explaining cell death from morphological, biochemical, and functional perspectives [21,22]. ACD refers to catastrophic cell death under natural conditions [23]. As an autonomous cell death process, PCD involves intracellular suicide pathways controlled by strict genetic mechanisms that maintain a stable internal environment, critical in response to inflammation, infection, and injury [24]. Based on its different mechanisms [25], PCD has two major categories: apoptosis and non-apoptotic programmed cell death (NAPCD). NAPCD includes autophagy, ferroptosis, pyroptosis, immunogenic cell death, mitochondrial catastrophe, necroptosis, and anoikis [26]. As research on NAPCD continues to advance, we have found that NAPCD has excellent potential in the pathogenesis and diagnosis of various diseases, such as various malignant tumors and even in benign diseases such as hearing impairment [22,27,28,29,30].

As the most common form of NAPCD [31], autophagy refers to cellular self-digestion mediated by lysosomal hydrolases to maintain normal intracellular and tissue homeostasis when cells lack nutrients or are affected by inflammation [32]. Ferroptosis is also a form of NAPCD, characterized by lipid peroxidation damage to the cell membrane and the production of iron ion-dependent lipid reactive oxygen species [33]. Pyroptosis is a recently proposed NAPCD-dependent activation of cellular inflammation through inflammatory forms of regulated cell death [34]. It can lead to PCD under various conditions [35]. Immunogenic cell death (ICD) is a new type of NAPCD [36] that activates adaptive immune responses in an immunoreactive environment [22]. ICD is caused by certain chemotherapeutic agents, lytic virus, physical chemotherapy, photodynamic therapy, and radiotherapy, which induce cell death by activating the immune system of an immunocompetent host [37]. Other types of NAPCD, such as mitotic catastrophe, necroptosis, and anoikis, have been less reported in thyroid-related diseases [38,39].

In conclusion, NAPCD provides a new frontier in the pathogenesis and treatment of the disease. In this paper, we have reviewed the progress of research on NAPCD in benign and malignant thyroid diseases, including efficacy prediction, drug resistance, therapeutic targets, and the relationship between different NAPCDs for clinical diagnosis and treatment.

## 2. Autophagy and Thyroid Disease

Autophagy is the most common type of NAPCD, and as a highly conserved physiological process, it removes damaged organelles and abnormal proteins through lysosomal degradation [40]. Autophagy is implicated in various pathological and physiological processes, including neurodegenerative diseases [41], the maintenance of intracellular homeostasis [42], inflammation [43], and cancer [44]. Autophagy is widely believed to have a dual effect on cells. On the one hand, it supports cell survival by adjusting the physiologically relevant mechanisms required to support cell proliferation and survival and maintains a stable internal environment [45,46]. On the other hand, it regulates cell death by regulating cellular autophagy-related mechanisms [47,48,49].

**Figure 1:**
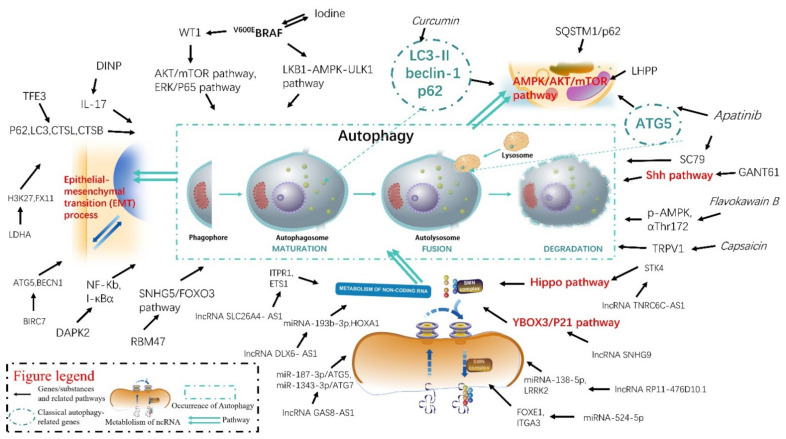
The mechanisms of autophagy in thyroid cancer.

A comprehensive understanding of the mechanisms of autophagy in the pathogenesis and progression of thyroid diseases will help determine the appropriate timing, effective therapeutic targets, and provide innovative ideas for diagnosing and treating thyroid-related diseases. We attempted to elucidate the changes in autophagy-related gene or RNA expression, therapeutic targets, and natural substances or their extracts in TC, which help us to better understand the autophagy mechanism in TC occurrence and its development comprehensively (Figure 1).

### 2.1. The Role of Genes and Autophagy in Thyroid Disease

Certain genes can significantly affect autophagy by triggering changes at the protein or RNA level, which may promote or inhibit the development of thyroid-related diseases. By elucidating the genes that affect the occurrence and development of thyroid-related diseases, we have a deeper understanding of the pathogenesis of thyroid diseases, and also laid the foundation for us to subsequently explore target molecules that can help in the early diagnosis of diseases and the development of targeted drugs.

BRAF mutations are the most common genetic lesions in thyroid tumors, with an incidence of 45% in PTC and 25% in ATC [50]. Currently, ^V600E^BRAF mutations are considered highly-specific diagnostic genetic markers for PTC, and ^V600E^BRAF is closely related to the development and metastasis of PTC [51]. ^V600E^BRAF mutants activate marker pathways and promote cancer progression in PTC [52] and Wilms’s tumor 1 (WT1), encoded as a transcription factor located on chromosome 11p13 [53]. Targeting the ^V600E^BRAF mutant is an effective treatment for PTC, and BRAF activation of WT1 promotes the growth of PTC and regulates autophagy and apoptosis [54].

Adenosine monophosphate-activated protein kinase (AMPK) regulates cellular metabolism as an energy sensor by mediating the insulin pathway [55,56]. Autophagy can be modulated through the AKT/AMPK/mTOR pathway [57,58]. Two studies have identified the role of autophagy and the AKT/AMPK/mTOR pathway in thyroid tumors. Sequestosome 1 (SQSTM1), also known as p62, is a vital gene in autophagy that regulates intracellular protein degradation [59]. SQSTM1 has been suggested to regulate autophagy via the AKT/AMPK/mTOR signaling pathway to trigger autophagy and promote the growth of papillary thyroid cancer cells [60]. Some researchers have suggested that SIRT6 can inhibit the glucose transporter protein 1 (GLUT1) through autophagy-mediated degradation, thereby suppressing the Warburg effect that affects tumor growth and development [60]. Furthermore, as a histidine phosphatase, phospholysine phosphohistidine inorganic pyrophosphate phosphatase (LHPP) is an antitumor factor [61,62]. LHPP similarly inhibits papillary TC cell growth and migration by regulating the AKT/AMPK/mTOR signaling pathway and triggering autophagy [57]. In addition, the sonic hedgehog (Shh) pathway has been implicated in autophagy in TC. The Shh pathway is highly activated in various malignancies and plays an essential role in tumor development [63]. Inhibition of the hedgehog pathway has been suggested to activate TGF-β-activated kinase (TAK1), which inhibits the apoptosis of thyroid tumor cells by inducing autophagy onset [64].

Epithelial-mesenchymal transition (EMT) appears early in the tumor metastasis process and plays a crucial role in mediating the development of aggressive tumor phenotypes. EMT is a multistage process in which cells lose their epithelial properties and undergo significant changes in morphology, adhesion, and migration capacity [65]. The core features of the EMT include decreased adhesion and increased motility [66]. Baculoviral IAP repeat-containing 7 (BIRC7) has been suggested to promote epithelial-mesenchymal transition and metastasis in papillary TC by inhibiting autophagy [67]. Lactate dehydrogenase A (LDHA) is an important enzyme involved in the Warburg effect. This leads to the formation of an acidic microenvironment in the tumor that promotes EMT and metastasis [68]. LDHA has been suggested to regulate autophagy to promote metastasis and tumorigenesis in PTC by inducing EMT gene transcription [68].

In addition, several other genes have been reported to influence the occurrence and development of thyroid disease through the autophagic pathway. FOXO3 belongs to the Forkhead Box (FOX) family of transcription factors [69]. The aberrant activation of FOXO3 has been extensively studied in cancer development and progression [70]. FOXO3 can promote autophagy through the transcriptional activation of autophagy-related genes, suggesting that FOXO3 can serve as a marker of autophagy [71]. The RNA binding motif protein 47 (RBM47)/small nucleolar RNA host gene 5 (SNHG5)/FOXO3 axis inhibits PTC cell proliferation by activating autophagy [72]. Furthermore, as another critical transcription factor of the FOX family [73], FOXK2 was found to promote the proliferation of PTC cells through the downregulation of autophagy [74]. BIRC7 is a newly identified member of the IAP family that is largely absent in normal tissues but is expressed at elevated levels in a range of tumor types [75]. The overexpression and expression of BIRC7 in tumors are related to the increased resistance to chemotherapy and decreased patient survival [76]. BIRC7 is considered a potential new target for thyroid tumor therapy. As a regulator of autophagy and lysosomal biogenesis, the transcription factor E3 (TFE3) belongs to the microphthalmia/transcription factor E (MiT/TFE) family, located on the short arm of the X chromosome 11.22 [77]. TFE3 can contribute to the invasion and metastasis of PTC by regulating autophagy [78]. As a member of the Ca^2+^/calmodulin-regulated serine/threonine kinases family, death-associated protein kinase 2 (DAPK2) is a tumor suppressor that affects various cellular activities, including cellular immune function and cell death. Recent studies have shown that DAPK2 can participate in autophagy and activate NF-κB through the autophagy-dependent degradation of IκBα, affecting TC development and progression [79].

With continuous advances in high-throughput genome sequencing technology, we have observed that 90% of the human genome could undergo transcription [80]. However, not all RNAs can be translated into proteins [81]. We refer to genes that do not directly encode proteins as non-coding RNAs (ncRNAs). ncRNAs play vital roles in human disease progression by regulating gene expression [82]. ncRNAs can contribute to mRNA degradation and protein translation failure by mediating post-transcriptional gene silencing [83]. In addition, ncRNAs also remodel chromatin structure by altering heterochromatin formation [84], thus enhancing or repressing gene expression [83]. These regulations can affect cellular function and help maintain homeostasis in vivo [85]. Several ncRNAs influence the onset of thyroid-related diseases through autophagy in thyroid-related diseases.

Long non-coding RNAs (lncRNAs) are classical ncRNAs: a heterogeneous family of RNA molecules greater than 200 nucleotides in length. They have gained widespread attention for their potential roles in organism development and disease [86]. Aberrant lncRNA expression has been observed in various cancers [87]. LncRNAs in PTC carcinogenesis and development play a vital role in thyroid disease development through autophagic mechanisms. LncRNA SLC26A4-AS1 can inhibit PTC progression by recruiting ETS1 to promote inositol 1,4,5-trisphosphate receptor type 1 (ITPR1)-mediated autophagy [88]. The lncRNA distal-less homeobox 6 antisense RNA 1 (DLX6-AS1) can interact with microRNA-193b-3p to inhibit TC progression by suppressing homeobox A1 (HOXA1) and enhancing autophagy and apoptosis in TC cells [89]. The lncRNA TNRC6C-AS1 promotes serine/threonine kinase 4 (STK4) methylation and inhibits TC cell autophagy through the hippo signaling pathway [90]. Activating transcription factor-2 (ATF2)-inducible lncRNA growth arrest-specific 8 (GAS8)-AS1 promotes TC cell progression by targeting miR-1343-3p/ATG7 and miR-187-3p/ATG5 axes to promote autophagy in TC cells [91]. LncRNA RP11-476D10.1 can enhance autophagy in PTC cells while inhibiting their proliferation through microRNA-138-5p-dependent inhibition of LRRK2 [92]. Furthermore, SNHG9, an exosome-rich lncRNA in PTC cells, can inhibit autophagy through the YBOX3/P21 pathway in normal thyroid epithelial cells [93].

MicroRNAs (miRNAs) are a class of non-coding RNAs of 18–24 nucleotides in length involved in various physiological processes. miRNAs are involved in target gene regulation by inhibiting protein production through binding to complementary mRNAs and are tissue- and stage-specific [94]. miRNA-524-5p inhibits the progression of PTC cells by targeting FOXE1 and ITGA3 in the cellular autophagy and recycling pathways [95].

### 2.2. The Role of Inhibitors, Substances, and Autophagy in Thyroid-Related Diseases

Natural products have been used as alternative therapies for various diseases, including inflammation and cancer. They have received attention from the academic community because of their cost and relatively few side effects [96,97].

Curcumin is widely used in traditional medicine and is a phytochemical isolated from the spice turmeric (Curcuma longa) [98]. Curcumin, used primarily as an adjuvant in cancer treatment, is undergoing extensive clinical trials resulting in favorable results [99,100,101]. Curcumin reportedly induces autophagic death in human thyroid cancer cells [102].

ATC is the most lethal subtype of TC. Lacking of sodium iodide synthetics (NIS) is a characteristic of the highly dedifferentiated state of ATC115. Therefore, radioactive iodine (RAI) therapy, which relies on iodine uptake by NIS channels, cannot be applied to ATC patients [103,104]. However, severe side effects can develop in TC patients treated with chemotherapy, such as high blood pressure, hypocalcemia, and hypoalbuminemia and drug resistance [105]. This means that finding new targeted drugs or exploring multidrug combinations may be a new research direction for the treatment of TC. As novel orally targeted TKIs, apatinib can the inhibit vascular endothelial growth factor receptor 2 (VEGFR2) with high selectivity. Importantly, apatinib has shown promising efficacy in few patients with radioiodine-refractory differentiated TC [106]. Apatinib has been shown to inhibit proliferation and induce autophagy through the PI3K/Akt/mTOR signaling pathway in human PTC cells [107]. Apatinib has been suggested to induce autophagy by downregulating p-AKT and p-mTOR signaling through the AKT/mTOR pathway in human ATC cells [108]. It has been reported that apatinib induces autophagy and apoptosis in human ATC cells by blocking the Akt/GSK3/ANG pathway to inhibit angiogenesis in mesenchymal TC [109]. In addition, as a natural alkaloid derived from Capsicum spp. [110], Capsaicin (trans-8-methyl-N-vanillyl-6-nonenamide, CAP) is a natural alkaloid [110], which inhibits the stemness of mesenchymal TC cells by activating autophagic lysosomal-mediated degradation of OCT4A [111]. The action of CAP on ATC helps us explore new ATC-targeting drugs [108,112,113,114].

Herbal plants have a long history of clinical application in China. *Prunella vulgaris* L. (PV) is a traditional herbal medicine used in ancient China to treat thyroid disorders [115]. Under environmental stress conditions, AMPK, the mammalian target of rapamycin (mTOR), and unc-51-like autophagy-activated kinase 1 (ULK1) constitute a pathway that can initiate cellular autophagy [116]. PV aqueous extract can inhibit the growth of papillary thyroid carcinomas through the induction of autophagy in vitro and in vivo, possibly due to being autophagy-mediated by the AMPK/ mTOR/ULK1 pathway [117].

In the past, the vast majority of the world’s population consumed iodized salt to reduce iodine deficiency disorders, leaving most of the population in a state of iodine excess [118,119]. High iodine levels may affect the occurrence of PTC through BRAF gene mutations [120]. ^V600E^BRAF can render PTC more susceptible to extrathyroidal infiltration and lymph node metastasis by aberrantly activating the BRAF/MEK/ERK (MAPK) signaling pathway [121]. High iodine levels are an important risk factor in the formation of ^V600E^BRAF mutation-associated tumors, followed by increased overexpression and activity of BRAF kinase [122]. Studies by several authors indicate that autophagy induced by BRAF kinase in PTC cells is involved in anti-apoptosis, and promotes proliferation and migration at high iodine concentrations [123].

In addition, some specific inhibitors, or biochemicals, may also affect thyroid-related diseases through the autophagic pathway. Di-isonylphthalate (DINP) has a wide range of applications in artificial leather and coated fabrics [124]. It has been suggested that DINP exacerbates autoimmune thyroid disease in Wistar rats by inhibiting autophagy via the activation of the Akt/mTOR pathway [125]. Furthermore, some researchers have suggested that the adenosine 5’-monophosphate-activated protein kinase-dependent mTOR pathway is involved in Flavokawain BLHPP (FKB)-induced autophagy in TC cells [126].

### 2.3. Role of Drug Resistance and Autophagy in Thyroid Disease

Drug resistance is a classic theme in cancer therapy, and its development may be based on multiple mechanisms [127,128,129]. Autophagy, however, is a new cause of drug resistance in tumors that has been proposed in recent years [130,131,132,133,134]. Many therapeutic regimens induce cytoprotective autophagy, rendering cancer cells less sensitive to these drugs. Our exploration of the mechanisms of autophagy helps us uncover new targets, which may lead to breakthroughs in drug resistance to anticancer therapies [135].

The presence of ^V600E^BRAF mutations is strongly associated with rapid TC progression, extrathyroidal infiltration, lymph node metastasis, and tumor recurrence [50,136]. Several ^V600E^BRAF inhibitors (BRAFi), such as vemurafenib and dabrafenib, have been marketed for approval. Targeting ^V600E^BRAF therapies have resulted in benefits to many patients [137]. However, a significant proportion of patients still develop resistance to BRAFi and progress to more advanced diseases. Melanoma is considered one of the most aggressive forms of skin cancer and the use of BRAF inhibitors, such as vemurafenib and dabrafenib, is revolutionizing the treatment of melanoma. Unfortunately, the duration of response to these drugs is limited due to the development of acquired resistance [138]. ^V600E^BRAF in TC cells inhibition has been shown to induce cytoprotective autophagy via the AMPK-ULK1 pathway [139]. This provides a deeper insight into the mechanisms of resistance to BRAFi.

## 3. Ferroptosis and Thyroid Disease

Ferroptosis is a type of NAPCD that relies on the continuous accumulation of lipid peroxides in the cell membrane, which ultimately leads to cell death [140]. The ferroptosis pathway is responsible for reducing lipid peroxides, mediated by the inactivation of glutathione peroxidase 4 (GPX4) [140]. In recent years, a growing number of studies have shown that ferroptosis is strongly related to cancer development and progression and opens up new possibilities for cancer therapy [141]. Targeting ferroptosis is an emerging anticancer strategy [142]. In previous studies from our team, we found that ferroptosis plays an important role in thyroid tumors [143].

A recent study examined several genes associated with ferroptosis that may influence immune infiltration and progression of TC, including the arachidonic acid 5-lipoxygenase-activating protein (ALOX5AP), B-cell CLL/lymphoma 3 (BCL3), and apolipoprotein E (APOE) [144]. Ferroptosis-associated ALOX5AP, BCL3, and APOE gene polymorphisms have been associated with TC risk [145]. These results help to better understand the relationship between TC susceptibility and genetic polymorphisms of ferroptosis-related genes.

As an essential dietary vitamin derived from fruits and vegetables, vitamin C can protect healthy cells from oxidative damage and act as a scavenger of free radicals in the body [146]. Vitamin C has been shown to induce ferroptosis in mesenchymal TC cells through ferritin phage activation [147]. This suggests that finding genes related to ferroptosis or autophagy may provide new targets for ATC therapy.

## 4. Pyroptosis and Thyroid Disease

Pyroptosis is a NAPCD that has only recently begun to attract the attention of scientists. Pyroptosis is believed to exist principally as a defense against pathogens by triggering an antimicrobial response through the release of immunogenic cellular content, including damage-associated molecular patterns (DAMPs) and inflammatory cytokines, which can lead to programmed cell death in various contexts [148,149]. Unlike other NAPCDs, cellular pyroptosis has a complex effect on the microenvironment. On the one hand, pyroptosis can affect the tumor immune microenvironment by affecting immune cells; on the other hand, many inflammatory factors are released during pyroptosis as normal cells are stimulated [150,151]. Pyroptosis was initially thought to be a primitive immune response to pathogens or their products and occurs in dendritic cells, monocytes, macrophages, and T cells [152]. The characteristic cell death pattern of pyroptosis also includes cell swelling, plasma membrane damage, and massive cytoplasmic leakage, particularly of IL-1β [153]. It has been reported that pyroptosis usually results from the activation of inflammatory cystathionase, resulting in gasdermin D protein hydrolysis cleavage [154].

Melittin, an isolated water-soluble peptide derived from honeybee venom, is used to alleviate chronic inflammation [155]. Apatinib, in the presence of melittin, induces recruitment and activation of inflammatory vesicles and leads to pyroptosis and enhanced antitumor effects of apatinib [156]. This suggests that apatinib in mesenchymal or invasive TC shows promising therapeutic benefits. Furthermore, low-dose apatinib synergistically achieves comparable therapeutic potential with melittin, thereby reducing adverse events. The positive feedback modulation may improve the therapeutic efficacy of antiangiogenic targeted agents, offering new prospects for targeted therapy [156].

AIT is a classic, organ-specific autoimmune disease. Epidemiological investigations have shown that environmental triggers and genetic susceptibility contribute to decreased tolerance and disease progression [157]. Cytokine secretion and release from thyroid follicular cells are mediated by enhanced AIM2, NLRC4, and NLRP1, and NLRP3 inflammasomes are related to autoimmune thyroiditis [158].

Hashimoto’s thyroiditis (HT) is a chronic form of autoimmune thyroiditis. The main manifestations of HT are autoimmune hypothyroidism and lymphocyte infiltration of the thyroid tissue [15]. Excessive iodine intake is a major risk factor for HT [159]. In the NOD-H-2h4 mouse model (spontaneous autoimmune thyroiditis model) [160], excessive iodine induces thyroid follicular cell scorch death and the production of unbalanced reactive oxygen species (ROS) in a mouse model, thereby inducing autoimmune thyroiditis via ROS [161]. This reveals a new cellular mechanism of abnormal cell scorching death in HT, thus contributing to our understanding of the mechanisms involved in the occurrence of scorching death and providing further insight into the HT mechanism.

Subacute thyroiditis (SAT) is a self-limiting inflammatory thyroid disease [162]. SAT is caused by the destruction of thyroid follicles, leading to the leakage of stored colloids, which results in thyrotoxicosis and elevated sedimentation rate with discomfort and fever [163]. For many years, SAT has been a rare disease that has been treated with NSAIDs or corticosteroids [164]. In recent years, there has been hope to devise a novel approach to analgesia, antiviral therapy, inflammation reduction, and the use of hormones without hormone dependence. Lidocaine is the original antiarrhythmic drug [165]. Some researchers have suggested that lidocaine treats subacute thyroiditis by inhibiting the pyroptosis pathway to inhibit adenovirus-induced apoptosis of thyroid follicular epithelial cells [166].

## 5. Other Non-Apoptotic Cell Death Mechanisms and Thyroid Disease

ICD is another NAPCD induced by certain chemotherapeutic agents, lytic viruses, physical chemotherapy, photodynamic therapy, and radiation therapy [37].

The coatomer protein complex zeta 1 (COPZ1) is involved in the retrograde transport of proteins in the endoplasmic reticulum Golgi secretory pathway [167] and lipid homeostasis [168]. COPZ1-deficiency cells have been found to initiate IFN/viral mimicry responses, and ICD, in turn, exacerbates inflammation and cell death. The link between ICD and the type-I interferon pathway is well established [169]. COPZ1 deficiency triggers type-I IFN responses and immunogenic cell death in thyroid tumor cells [170]. Immunotherapy, represented by immune checkpoint blockade, has changed the cancer treatment paradigm. The immune co-inhibitory receptor (CIR) and its corresponding ligands are essential components of the tumor microenvironment [171]. T-cell immunoglobulin and mucin-domain containing-3 (TIM-3), T-cell immunoglobulin and ITIM domain (TIGIT), lymphocyte activation gene-3 (LAG-3), cytotoxic T-lymphocyte antigen 4 (CTLA-4), and Protein-1 (PD-1) are considered to be the major immune CIRs and the most promising immunotherapeutic targets in cancer therapy [172].

MTC is a relatively rare malignant neuroendocrine tumor that exhibits aggressive clinical progression [173]. In a large cohort study of MTC, positive TIGIT, LAG-3, CTLA-4, PD-1, and TIM-3 expression was detected in 6 (3.0 %), 6 (3.0 %), 25 (12.5 %), 27 (13.5 %), and 96 (48.0%) patients, respectively, with a positive correlation between TIM-3, PD-1, and CTLA-4 expression. This suggests that TIM-3, CTLA-4 positivity, and PD1/PD-L1 co-positivity may be potential immune features related to structural tumor recurrence [174].

## 6. Summary

Thyroid disorders, a hot spot among endocrine-related diseases, have been extensively studied in recent years. We have observed that NAPCD has excellent potential for diagnosing and managing thyroid diseases. In our study, we have reviewed recently published studies related to NAPCD and thyroid-related diseases to further elucidate the relationship between autophagy, ferroptosis, pyroptosis, ICD, and the development and progression of thyroid-related diseases (Table 1). These studies have laid the theoretical foundation for clarifying the mechanisms of disease development, searching for new therapeutic agents, identifying drug resistance mechanisms, and exploring targets. With the continuous exploration of the role of NAPCD in diseases, we have a better understanding of benign and malignant thyroid diseases. NAPCD plays a distinct role in the pathogenesis of thyroid tumors. Its use to effectively inhibit cancer cell proliferation and achieve precise treatment of thyroid-related diseases deserves further study by the academic community. In addition, we found that NAPCD may be associated with tumor drug resistance, which may help provide a new research direction for us to explore the mechanism of reversing drug resistance. Furthermore, elucidating the role of NAPCD in thyroid disease will help us explore novel drugs and targets. Consequently, exploring the underlying mechanisms in thyroid tumors and inflammation-related diseases has helped us better understand the self-mechanisms of NAPCD.

## Figures and Tables

**Table 1:** The characteristic summary of NAPCD in thyroid cancer.

		Drugs or Inducers	Disease	Key Factor	Pathway	In Vivo/In Vitro Experimental Validation	Reference
**autophagy**	**genes**	V600EBRAF	papillary thyroid cancer	WT1	AKT/mTOR pathway, ERK/P65 pathway	In vivo and in virto	[57]
		SQSTM1/p62	papillary thyroid cancer	LC3-II	AMPK/AKT/mTOR pathway	In vivo and in virto	[63]
		V600EBRAF	papillary thyroid cancer		LKB1-AMPK-ULK1 pathway	In vivo and in virto	[113]
		SIRT6	papillary thyroid cancer	GLUT1	Warburg effect	In vivo and in virto	[63]
		BIRC7	papillary thyroid cancer	ATG5, BECN1	EMT	In vivo and in virto	[70]
		GANT61	anaplastic thyroid cancer	LC3-II, p62, TAK1, JNK, AMPK	Shh pathway	In vitro	[67]
		LDHA	papillary thyroid cancer	H3K27, FX11	EMT	In vivo and in virto	[71]
		FOXK2	papillary thyroid cancer	ULK1, VPS34, FOXO3		In vitro	[78]
		RBM47	papillary thyroid cancer	LC3-II, p62	SNHG5/FOXO3 pathway	In vivo and in virto	[76]
		TFE3	papillary thyroid cancer	P62, LC3, CTSL, CTSB		In vitro	[82]
		DAPK2	papillary thyroid cancer	NF-Κb, I-κBα		In vivo and in virto	[83]
	**compounds**	Capsaicin	anaplastic thyroid cancer	TRPV1		In vitro	[46]
		Prunella vulgaris L.	papillary thyroid cancer	LC3-II, beclin-1, p62	AMPK/mTOR/ULK1 pathway	In vivo and in virto	[49]
		Apatinib	papillary thyroid cancer	ATG5	PI3K/Akt/mTOR pathway	In vivo and in virto	[101]
		Apatinib	anaplastic thyroid cancer	SC79	AKT/mTOR pathway	In vivo and in virto	[103]
		Iodine	papillary thyroid cancer	V600EBRAF	BRAF/MEK/ERK (MAPK) pathway	In vitro	[108]
		DINP	autoimmune thyroid disease	IL-17	Akt/mTOR pathway	In vivo	[112]
		Flavokawain B	thyroid cancer	p-AMPK, αThr172	AMPK/mTOR pathway	In vitro	[113]
		Curcumin	papillary thyroid cancer	LC3-II, beclin-1, p62	AMPK/AKT/mTOR pathway	In vitro	[41]
		LHPP	papillary thyroid cancer		AMPK/AKT/mTOR pathway	In vivo and in virto	[60]
	**ncRNA**	lncRNA SLC26A4- AS1	papillary thyroid cancer	ITPR1, ETS1		In vitro	[92]
		lncRNA DLX6- AS1	papillary thyroid cancer	microRNA-193b-3p, HOXA1		In vivo and in virto	[93]
		lncRNA TNRC6C-AS1	papillary thyroid cancer	STK4	Hippo pathway	In vivo and in virto	[94]
		lncRNA GAS8-AS1	papillary thyroid cancer	ATF2	miR-187-3p/ATG5, miR-1343-3p/ATG7	In vivo and in virto	[95]
		lncRNA RP11-476D10.1	papillary thyroid cancer	microRNA-138-5p, LRRK2		In vitro	[96]
		lncRNA SNHG9	papillary thyroid cancer	SNHG9	YBOX3/P21 pathway	In vitro	[97]
		MicroRNA-524-5p	papillary thyroid cancer	FOXE1, ITGA3		In vitro	[99]
							
**Ferroptosis**	**genes**	APOE	thyroid cancer			In vivo	[126]
		BCL3	thyroid cancer			In vivo	[126]
		ALOX5AP	thyroid cancer			In vivo	[126]
	**compounds**	Vitamin C	anaplastic thyroid cancer	GPX4		In vitro	[128]
	**ncRNA**	Circ_0067934	thyroid cancer	miR-545-3p/SLC7A11		In vitro	[130]
							
**Pyroptosis**	**genes**	NLRP3	autoimmune thyroiditis			In vivo	[137]
		NLRP1	autoimmune thyroiditis			In vivo	[137]
		NLRC4	autoimmune thyroiditis			In vivo	[137]
		AIM2	autoimmune thyroiditis			In vivo	[137]
	**compounds**	Iodine	hashimoto’s thyroiditis	ROS		In vivo	[141]
		lidocaine	subacute thyroiditis			In vitro	[146]
		Melittin	anaplastic thyroid cancer	apatinib		In vitro	[135]
							
**Immunogenic cell death**	**genes**	COPZ1	papillary thyroid cancer	type I IFN		In vitro	[151]
		TIM-3	medullary thyroid carcinoma			In vivo	[155]
		PD-1	medullary thyroid carcinoma			In vivo	[155]
		CTLA-4	medullary thyroid carcinoma			In vivo	[155]
		LAG-3	medullary thyroid carcinoma			In vivo	[155]
		TIGIT	medullary thyroid carcinoma			In vivo	[155]

## Data Availability

Data sharing not applicable.

## Abbreviations

non-apoptotic programmed cell death (NAPCD); thyroid cancer (TC); papillary thyroid cancer (PTC); follicular thyroid cancer (FTC); medullary thyroid cancer (MTC); radioactive iodine (RAI, 131I); tyrosine kinase inhibitors (TKIs); autoimmune thyroiditis (AIT); Graves’ disease (GD); Hashimoto’s thyroiditis (HT); programmed cell death (PCD); accidental cell death (ACD); immunogenic cell death (ICD); Wilms’s tumor 1 (WT1)2; Sequestosome 1 (SQSTM1); glucose transporter protein 1 (GLUT1); phospholysine phosphohistidine inorganic pyrophosphate phosphatase (LHPP); sonic hedgehog (Shh); TGF-β-activated kinase (TAK1); Epithelial-mesenchymal transition (EMT); Baculoviral IAP repeat-containing 7 (BIRC7); Lactate dehydrogenase A (LDHA); Forkhead Box (FOX); RNA binding motif protein 47 (RBM47); small nucleolar RNA host gene 5 (SNHG5); transcription factor E3 (TFE3); microphthalmia/transcription factor E (MiT/TFE); death-associated protein kinase 2 (DAPK2); non-coding RNAs (ncRNAs); long non-coding RNAs (lncRNAs); distal-less homeobox 6 antisense RNA 1 (DLX6-AS1); homeobox A1 (HOXA1); serine/threonine kinase 4 (STK4); Activating transcription factor-2 (ATF2); growth arrest-specific 8 (GAS8); microRNAs (miRNAs); Prunella vulgaris L. (PV); adenosine monophos-phate-activated protein kinase (AMPK); unc-51-like autoph-agy-activated kinase 1 (ULK1); sodium iodide synthetics (NIS); radioactive iodine (RAI); vascular endothelial growth factor receptor 2 (VEGFR2); Di-isonylphthalate (DINP); Flavokawain BLHPP (FKB); glutathione peroxidase 4 (GPX4); arachidonic acid 5-lipoxygenase-activating protein (ALOX5AP); B-cell CLL/lymphoma 3 (BCL3); apolipoprotein E (APOE); damage-associated molecular patterns (DAMPs); Hashimoto’s thyroiditis (HT); reactive oxygen species (ROS); Subacute thyroiditis (SAT); coatomer protein complex zeta 1 (COPZ1); T-cell immunoglobulin and mucin-domain containing-3 (TIM-3); T-cell immunoglobulin and ITIM domain (TIGIT); lymphocyte activation gene-3 (LAG-3); cytotoxic T-lymphocyte antigen 4 (CTLA-4); Protein-1 (PD-1).
